# Differential Expression Pattern of THBS1 and THBS2 in Lung Cancer: Clinical Outcome and a Systematic-Analysis of Microarray Databases

**DOI:** 10.1371/journal.pone.0161007

**Published:** 2016-08-11

**Authors:** Tzu-Yang Weng, Chih-Yang Wang, Yu-Hsuan Hung, Wei-Ching Chen, Yi-Ling Chen, Ming-Derg Lai

**Affiliations:** 1 Department of Biochemistry and Molecular Biology, College of Medicine, National Cheng Kung University, Tainan, Taiwan; 2 Institute of Basic Medical Sciences, College of Medicine, National Cheng Kung University, Tainan, Taiwan; 3 Center for Infectious Diseases and Signal Research, National Cheng Kung University, Tainan, Taiwan; 4 Department of Senior Citizen Services Management, Chia Nan University of Pharmacy and Science, Tainan, Taiwan; Institute of Biomedical Sciences, TAIWAN

## Abstract

Thrombospondin 1 and thrombospondin 2 (THBS1 and THBS2) share similar multifunctional domains, and are known to be antiangiogenic. However, the expression pattern of THBS1 and THBS2 is different, and the specific role of THBS2 in different subtypes of lung cancer remains largely unclear. To evaluate the significance of THBS1 and THBS2 in the development of lung cancer, the present study performed a microarray-based systematic-analysis to determine the transcript levels of thrombospondins and their relation to the prognosis in lung cancer. THBS1 was in general underexpressed in lung cancer; in contrast, mRNA levels of THBS2 were markedly overexpressed in a number of datasets of non-small cell lung carcinoma (NSCLC), including lung adenocarcinoma (AC) and squamous cell carcinoma. Similar expression pattern of THBS1 and THBS2 was verified in pulmonary AC cell lines with real-time PCR analysis. The survival of lung AC patients with high THBS2 mRNA expression levels was poorer than patients with low levels of expression of THBS2. In a microarray-based analysis, genes coexpressed with THBS1 or THBS2 were determined. Pulmonary AC patients with a high expression level of sevenTSHB1-coexpressed genes (CCL5, CDH11, FYB, GZMK, LA-DQA1, PDE4DIP, and SELL) had better survival rates than those with a low expression level. Patients with a high expression of seven TSHB2-coexpressed genes (CHI3L1, COL5A2, COL11A1, FAP, MXRA5, THY1, and VCAN) had poor survival rates. Downregulation of VCAN and THBS2 with shRNA inhibited the cell proliferation in the A549 cell line. In summary, THBS1 functions as a tumor suppressor in lung adenocarcinoma. However, THBS2 may play a double-edged role in the progression of lung AC, i.e. anti-angiogenic and oncogenic function. Further study on the mechanism underlying the activity of THBS2 is warranted to have further implications for cancer diagnosis and treatment of pulmonary AC.

## Introduction

Lung cancer is the leading cause of cancer mortality in the world in recent decades, accounting for about 20% of all cancer deaths in both men and women [1]. Histologically, there are two major types of lung cancer, non-small cell lung cancer (NSCLC) and small cell lung cancer, with 85% of cases due to NSCLC. NSCLC can be divided into three main subtypes: adenocarcinoma (AC, 40% of lung cancers), squamous cell carcinoma (SCC, 25–30% of lung cancers), and large cell carcinoma (10% of lung cancers). Overall, the 5-year survival rate for patients with NSCLC is less than 18%, and it is only about 7% for patients with small cell lung cancer [1]. Metastatic spread was reported in more than 70% of NSCLC patients with advanced-stage disease, with the metastases mainly affecting the brain, liver and bone sites. In all cases, the patients died within 18 months or soon after. Investigating changes in the tumor-associated microenvironment during cancer progression is important for targeted therapy and improvement of clinical outcomes in lung cancer [2]

Thrombospondins (THBSs or TSP) are secreted glycoproteins, with various functional domains involved in embryonic development, wound healing [3], angiogenesis [4], and inflammatory response [5, 6]. THBSs are subdivided into two subgroups: subgroup A and subgroup B. Subgroup A includes THBS1 and THBS2, which can form trimers. Subgroup B, which includes THBS3, THBS4, and THBS5 (also referred to as cartilage oligomeric matrix protein [COMP]), can form pentamers. A distinct feature of subgroup A is the presence of three thrombospondin type 1 (TSR) repeats, which interact with CD36 and beta-1 integrins. The interaction of the TSR domain and membrane CD36 in endothelial cells suppresses cell migration and induces apoptosis, which results in the inhibition of angiogenesis. Only THBS1 contains an RFK motif located between the first- and second-repeat of the TSR domain and responds to the activation of transforming growth factor-beta [7]. Except for THBS5/COMP, all THBS proteins contain an N-terminal domain as a signature motif and are involved in cell adhesion through binding with several receptors or ligands, such as calreticulin and integrins [8]. Five THBSs have a carboxy-terminal domain, which interacts mainly with CD47, in addition to at least three copies of epidermal growth factor-like domain (type 2 repeats) and several copies of calcium-binding domains (type 3 repeats) [9].

THBS1 is the most studied gene among the THBS family. It plays a functional role in inhibiting tumor growth, cell migration, and neovascularization; it also acts as an endogenous tumor suppressor by interacting with its receptors, CD36 and CD47, or activating transforming growth factor-beta signaling [10]. Decreasing levels of THBS1 in NSCLC were reported to be correlated with worse prognoses [11]. Several THBS1-based compounds are in development for cancer therapy [12]. On the other hand, the expression profile of another subgroup A member, THBS2, is variable in different types of cancers. The expression of THBS2was up-regulated in some types of cancers [13–15], but was down-regulated in other types of cancers [16, 17]. High levels of THBS2 and fibroblast growth factor-2 in the serum of NSCLC patients predicted poor survival rates [18]. In contrast, overexpression of THBS2 suppressed tumor growth in squamous cell carcinomas and Lewis lung carcinoma xenograft mouse tumor models [19]. A truncated-recombinant THBS2 protein inhibited tumor growth and angiogenesis *in vivo* [20].

Although THBS subgroup A members share many structural domains or functional motifs, there are few studies systematically evaluating their expression patterns or importance in human lung cancer using clinical microarray databases. The Oncomine cancer microarray database integrates gene expression data and clinical data, and contains 20 major cancers and over 4,700 microarray experiments [21]. The Kaplan–Meier plotter database integrates gene expression data and clinical data and contains information on 22,277 genes and their effects on survival in 2,437 lung cancer patients [22]. In the present study, both the Oncomine and Kaplan–Meier plotter databases were used to investigate whether the transcript levels of THBS1 and THBS2 were altered in lung and correlated with the clinical prognosis. In addition, the functional characteristics and molecular mechanism of THBS2 and its coexpressed genes were investigated in a systematic-analysis. The results may shed light on the role of THBS2 in the tumor microenvironment during the progression of lung AC. To the best of our knowledge, this is the first study to systematically evaluate the correlation between transcript levels of THBSs and clinical outcomes in lung cancer patients using a systematic-analysis.

## Methods

### Oncomine database analysis

**Fig 1:**
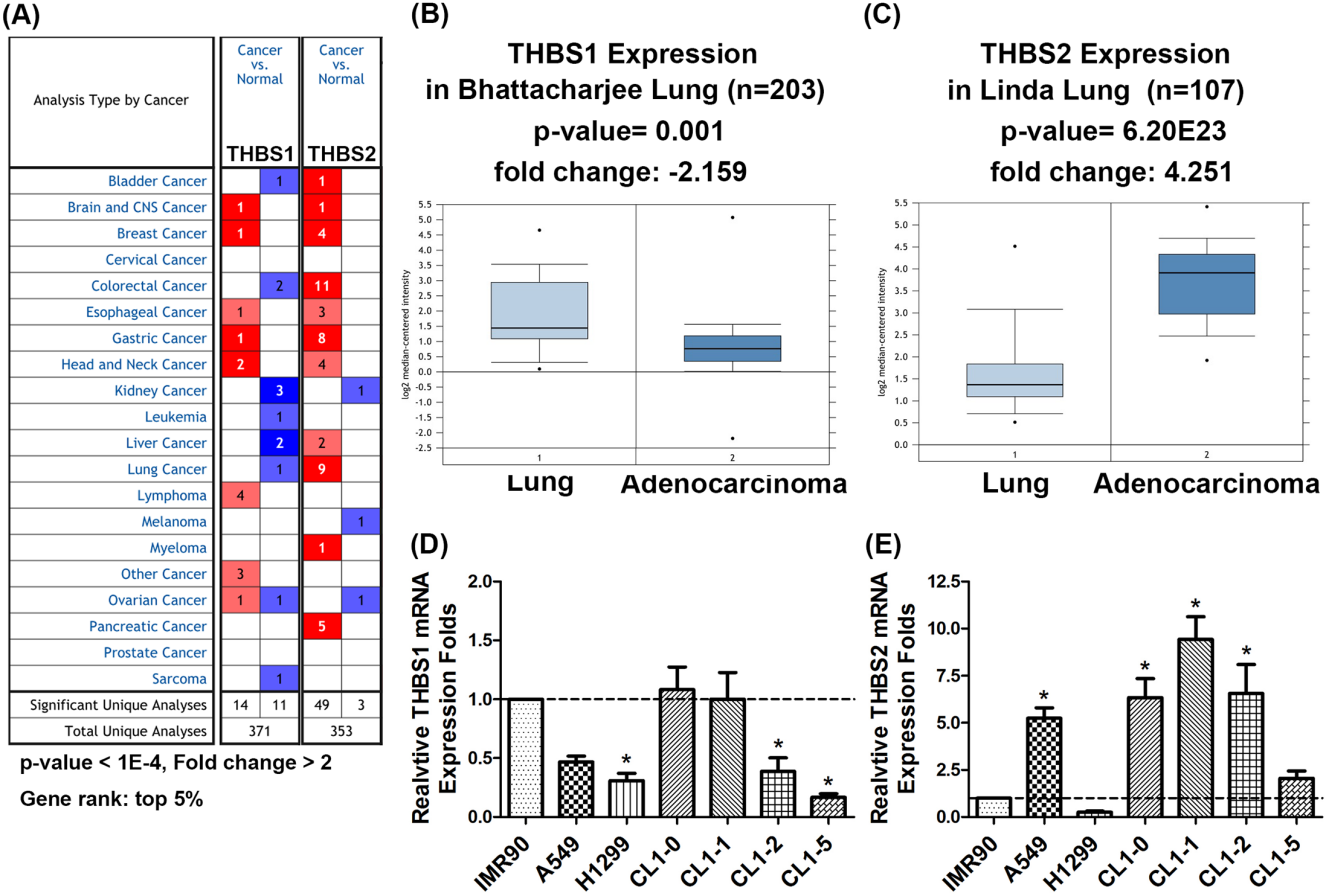
THBS2 was overexpressed in lung adenocarcinoma tissue. (A) Expressions of THBS1 and THBS2 in 20 common cancers were compared with corresponding normal tissues (Oncomine Database). The threshold of search criteria for datasets of cancer vs. normal analysis was a p-value <1E-4, a fold change >2, and a gene rank in top 5%. Analysis of (B) THBS1 and (C) THBS2 mRNA expression in lung normal tissue and lung AC tissue by using Oncomine database. Relative (D) THBS1 and (E) THBS2 expression of six human lung AC cell lines (A549, H1299, CL1-0, CL1-1, CL1-2 and CL1-5) compared with the mean value of a normal lung cell line (IMR90). THBS1, Thrombospondin 1. THBS2, Thrombospondin 2. AC, adenocarcinoma.* represent a *P* value < 0.05.

Analysis of THBSs expression change in common or selected cancer tissues was performed by using the online cancer microarray database Oncomine, (www.oncomine.org, Compendia biosciences, Ann Arbor, MI, USA) [21]. mRNA expression of clinical specimens of tumor and normal (cancer vs. normal) was compared and extracted between April 2015 and June 2016. The threshold search criteria used for the Fig 1 (gene summary view) in the study were a p-value < 1E-4, a fold change > 2 and a gene rank in top 5%. P-values and fold-changes presented in this study for differential expression analysis of THBS genes were calculated with Oncomine using a two-sided Student’s t-test and multiple testing corrections. To examine the THBS2 or THBS1 coexpressed genes, the Oncomine tool was utilized to conduct the coexpression analysis of the microarray datasets. Three datasets (Garber Lung, Gordon Lung, and Landi Lung) were selected for the coexpression analysis, with each dataset consisting of >50 AC samples. To explore the THBS2 coexpressed gene in breast and gastric cancer, three breast cancer datasets (Ivshina Breast, Minn Breast 2 and Schmidt Breast) and three gastric cancer datasets (Chen Gastric, DErrico Gastric and DErrico Gastric) were analyzed. The top 5% of genes within each dataset were selected by using the co-expression score. The genes that appeared in at least two of the three datasets were defined as THBS2 and THBS1 coexpressed genes.

### Kaplan–Meier plotter database analysis

The survival analysis and hazard ratio estimation of the expression values of THBS1, THBS2, and coexpressed genes in cancers were performed with a Kaplan–Meier plotter online database (www.kmplot.com), which contains information on 22,277 genes and their effects on survival in 2,437 lung cancer, 4,142 breast, and 1,065 gastric patients (June 2015) [22]. The hazard ratios (95% confidence intervals) and log rank *p*-values were also computed using the Kaplan–Meier plotter database. The hazard ratios were estimated using a Cox-proportional hazards model. The progression-free survival rate of the lung cancer patients was analyzed. The patient samples were split into two groups with the best cut-off and compared using the Kaplan–Meier plotter. The specific probes (JetSet best probes) analyzed for recognizing each gene are listed in all related tables.

### SurvExpress database analysis

The SurvExpress database was used to further compare the survival rates of individuals segregated according to the THBS2 expression levels within each risk group (Okayama Lung dataset, GSE31210) [23]. The risk groups generated from the SurvExpress database were based on the prognostic index (PI) [24], and were split by the ordered PI (higher values for higher risk) with equal number of samples in each group. The PI was computed using the expression levels and values obtained from Cox fitting [23].

### Gene ontology and pathway enrichment analysis

Gene ontology and pathway enrichment analysis were conducted to examine THBS2 coexpressed genes (S4 Table) by using the Database for Annotation, Visualization and Integrated Discovery (DAVID; http://david.abcc.ncifcrf.gov/) [25]. The categories GOTERM_BP_3, GOTERM_CC_2 and GOTERM_MF_3, were selected, and all options were set as defaults. The data listed in the table were with hit count >5, and p-value <0.001.

### Construction of the gene interaction network

The gene interaction network was constructed with network building tool in MetaCore (Thompson Reuters, New York, NY) as previously described [26]. The direct interaction of the identified THBS2 coexpressed gene list (S1 Table)was predicted and obtained from MetaCore software. Genes with no interactions were not shown in the network.

### Cell culture

The lung cancer cell lines were kind gifts from Dr. Pan-Chyr Yang (National Taiwan University, Taipei, Taiwan) [27]. CL1-0, CL1-1, CL1-2 and CL1-5 cells were grown in RPMI 1640 media (LONZA, Walkersville, MD, USA); A549 and H1299 cells were grown in Dulbecco's Modified Eagle Medium (GIBCO, Carlsbad, CA, USA) media. Both culture media were supplemented with 10% fetal bovine serum (GIBCO, Carlsbad, CA, USA) and100 U/ml penicillin and 100 mg/ml streptomycin (HYCLONE, Logan, UT, USA). Cells were maintained at 37°C in 5% CO_2_ incubator.

### Real-time PCR

Lysate of human normal lung cell line, IMR90, was a kind gift from Dr. Yi-Ching Wang. Total RNA was extracted from cells by using TRIzol (Invitrogen, Carlsbad, CA, USA). cDNA was synthesized using MMLV reverse transcriptase (Promega, Madison, WI, USA). The following GAPDH, THBS1 and THBS2 sense and antisense primers were used as previously described: THBS1 5′-TTG TCT TTG GAA CCA CAC CA-3′ and 5′-CTG GAC AGC TCA TCA CAG G-3′ [28]; THBS2 5′-CGT GGA CAA TGA CCT TGT TG-3′ and 5′-GCC ATC GTT GTC ATC ATC AG-3′ [29]; GAPDH 5′-AGC CAC ATC GCT CAG ACA C-3′ and 5′-GCC CAA TAC GAC CAA ATC C-3′; VCAN 5’-TCC TGA TTG GCA TTA GTG AAG-3’ and 5’-CTG GTC TCC GCT GTA TCC-3’. Real-time PCR was performed on a StepOne ™ real-time PCR instrument (Applied Biosystems, Foster City, CA, USA) using Fast SYBR Green Master Mix (Applied Biosystems). The cycling conditions were 10min at 95°C and 45 cycles at 95°C for 15s and 60°C for 60s. The 2^ΔΔCt^ method was used to calculate the relative RNA expression, which was normalized with GAPDH expression.

### RNA interference and lentivirus production

THBS2 shRNAs and VCAN shRNAs were obtained from the National RNAi Core facility (Academia Sinica, Taipei, Taiwan). The TurboFect transfection reagent (Thermo Fisher Scientific, Slangerup, Denmark) was used to generate the lentiviral particles according to the protocol provided from the National RNAi Core facility. The following target sequences were used: THBS2 shRNA-1, GTG CCT TCA GAG GAT AAA TAT; THBS2 shRNA-2, GTC TTC AAT GAA CGA GAC AAT; VCAN shRNA-1, GCC ACA GTT ATT CCA GAG ATT; and VCAN shRNA-2, GTG AAT TTC TCC GCA TCA AAT.

### Cell proliferation assay

After incubation with lentivirus for 24 hours, infected A549 cells were seeded in 96-well plates at a cell density of 5,000 cells per well and cultured for 48 hours. Cell proliferation was examined with WST-1 Cell Proliferation Reagent (Clontech, Mountain View, CA, USA). 10μL WST-1 reagent was added into culture wells and incubating for 1hr. Absorbance was measured at wavelength of 450 nm by using a scanning multi-well spectrophotometer.

### Statistical analysis

p-values and fold-changes for differential expression analysis of THBS genes generated form Oncomine database were calculated using a two-sided Student’s t-test and multiple testing corrections with Oncomine. Statistical analyses of the mRNA expression in real-time PCR experiments were performed by using One-Way ANOVA in GraphPad Prism 5 software. Statistical analyses of the cell proliferation assay were performed by using the t-test in GraphPad Prism 5 software. *P* values <0.05 were considered significant.

## Results

### The expression patterns of THBS1 and THBS2 were diverse in various types of cancer

To determine changes in THBS and THBS2 transcripts in clinical specimens of lung cancer and other cancers, the mRNA level of THBSs in various types of cancer was examined using the Oncomine cancer microarray database. Based on the data on gene summary views (neoplastic vs. normal tissue), THBS2 was significantly up-regulated in 11 of 20 common cancers (Fig 1A). It was overexpressed in colorectal (11 of 36 studies) (S1 Table), gastric (8 of 24 studies) (S2 Table), lung (9 of 37 studies) (Table 1 and S3 Table), and pancreatic cancer (5 of 12 studies) (S4 Table). In contrast, only a small number of studies reported that THBS1 was significantly up- (S2 Table) or down-regulated (Table 1, S1 and S3 Tables) in different types of cancer (Fig 1A). We next focused on investigating the changes in THBS1 and THBS2 mRNA expression in lung cancer subtypes. The mRNA level of THBS1 was significantly underexpressed in small cell lung cancer (a fold change of -5.923) (S3 Table). Analysis of the expression levels of THBS1 in the two main subtypes of NSCLC in the same microarray dataset revealed decreased mRNA levels of THBS1 in both lung AD (a fold change of -2.159) (Fig 1B and Table 1) and lung SCC (a fold change of -1.968) (S3 Table), although the data did not satisfy the threshold criteria set in this study. On the other hand, mRNA levels of THBS2 were markedly overexpressed in lung AC (an average fold change of 3.308, *n* = 6) (Fig 1C and Table 1) and lung SCC (an average fold change of 8.915, *n* = 3) (S3 Table). To verify this finding, the mRNA expression of THBS1 and THBS2 was examined in a normal lung cell line (IMR90) and six lung AC cell lines (A549 H1299, CL1-0, CL1-1, CL1-2, and CL1-5) using real-time PCR. Lower expression levels of THBS1 were observed in four lung AC cell lines compared with those in normal lung cell lines (Fig 1D). The expression level of THBS2 mRNA was increased (more than 2-fold) in the A549, CL1-0, CL1-1, CL1-2, and CL1-5 lines (Fig 1E). This evidence suggested that THBS1 and THBS2 may play opposite roles in NSCLC.

**Table 1:** mRNA expression levels of THBS1 and THBS2 in lung adenocarcinoma.

Gene	P-Value (Cancer/Normal)	Fold Change (Cancer/Normal)	Ranking (Top%)	Dataset	#Samples	Reference
THBS1	0.001	-2.159	14	Bhattacharjee	203	1
THBS2	6.20E-23	4.251	1	Landi	107	2
	2.69E-9	3.356	1	Su	66	3
	2.55E-19	3.307	1	Selamat	116	4
	2.83E-8	3.416	2	Stearman	39	5
	1.11E-13	3.965	2	Hou	110	6
	1.52E-11	4.551	1	Wei	50	7

All references in this table were listed in the S7 Table.

### High THBS2 mRNA levels were correlated with a poor prognosis

**Fig 2:**
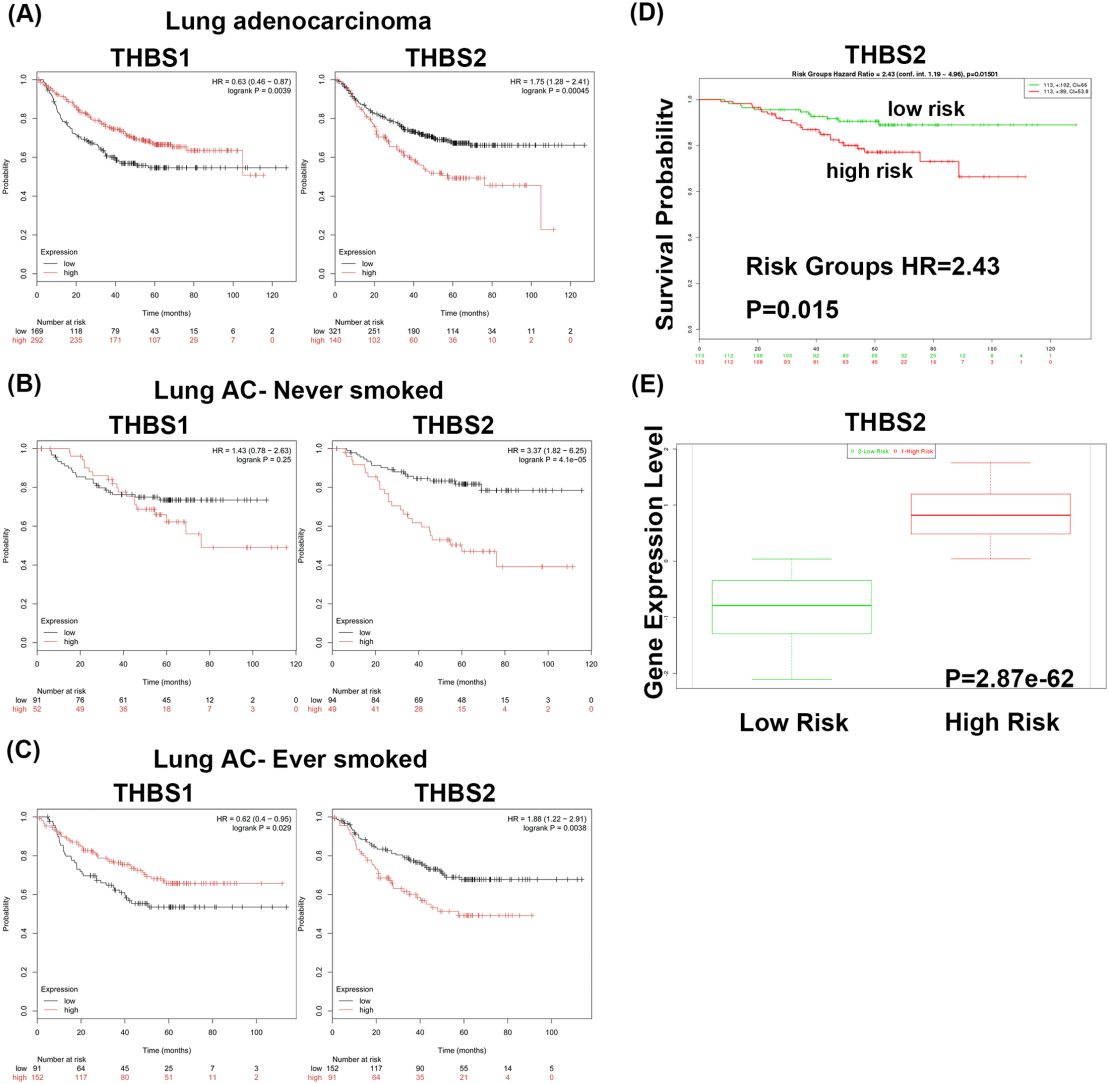
Expression level of THBS2 was associated to prognosis in patients with lung adenocarcinoma. (A)Kaplan-Meier survival curve (Kaplan-Meier plotter) showing the progression-free survival of THBS1 and THBS2 in lung adenocarcinoma. (B) Survival of lung AC patients who never smoked with THBS1 and THBS2 expression. (C) Survival of lung AC patients who were smokers with THBS1 and THBS2 expression. High and low expression in patients was represented in red and black, respectively. (D) Kaplan-Meier survival curve (SurvExpress, Okayama Lung dataset) showing the survival of lung AC patients with high or low THBS2 expression. High and low expressions were represented in red and green, respectively. (E) THBS2 expression levels with low and high risk groups generated from Okayama Lung dataset by using SurvExpress program. THBS, Thrombospondin. AC, adenocarcinoma.

To evaluate the association between THBS1 and THBS2 transcript levels and the survival of lung cancer patients, biomarkers were assessed using the Kaplan–Meier plotter. According to the results of the Kaplan–Meier plotter, the survival of pulmonary AC and pulmonary SCC patients with high THBS1 expression was better than that of patients with low expression (Fig 2A and Table 2). In contrast, the progression-free survival rates of pulmonary AC patients with high THBS2 expression were worse than those of patients with low expression (Fig 2A and Table 2). As tobacco smoking is strongly related to lung cancer development, the correlation between prognosis and THBS1 and THBS2 expression levels in patients, with and without a smoking history, were examined. The survival rates of AC lung cancer patients with high THBS2 expression were significantly worse both in the never-smoked (HR = 3.37) and ever-smoked (HR = 1.88) groups (Fig 2B and Table 2). In AC lung cancer patients with a history of smoking, the survival rates (HR = 0.62) of those with higher mRNA expression of THBS1 were better than those with lower mRNA expression of THBS1 (Fig 2C and Table 2). The associations of THBS2 expression with the risk of developing AC of the lung and the survival of those who developed the disease were further examined using another online biomarker validation tool, SurvExpress, in 226 primary lung ACs (Okayama Kohno Lung, GSE31210). This platform derives the risk groups and Kaplan–Meier curves with different expression levels. The expression level of THBS2 was correlated with poor survival rates (HR = 2.43, *P* = 0.015) (Fig 2D), and it was significantly increased in the high-risk group (Fig 2E). Together with the results of the systemic analysis, the results suggest that overexpression of THBS2 is a prognostic biomarker for poor survival rate in pulmonary AC.

**Table 2:** Analysis of THBS1 and THBS2 in lung cancer.

Gene	Log-rank p	Hazard ratio	Samples size	Probe
**Lung Adenocarcinoma**			461	
THBS1	0.0039**	0.63		201110_s_at
THBS2	0.0005***	1.75		203083_at
**-Only those never smoked**			143	
THBS1	0.246	1.43		201110_s_at
THBS2	4.1e-05***	3.37		203083_at
**-Exclude those never smoked**		243		
THBS1	0.029*	0.62		201110_s_at
THBS2	0.0038**	1.88		203083_at
**Squamous Cell Carcinoma**			141	
THBS1	0.0052**	0.37		201110_s_at
THBS2	0.108	0.66		203083_at

* indicated*p*<0.05.

** indicated *p*<0.01.

***indicated *p*<0.001.

### Specific genes coexpressed with THBS2 predicted a poor prognosis in pulmonary AC

**Fig 3:**
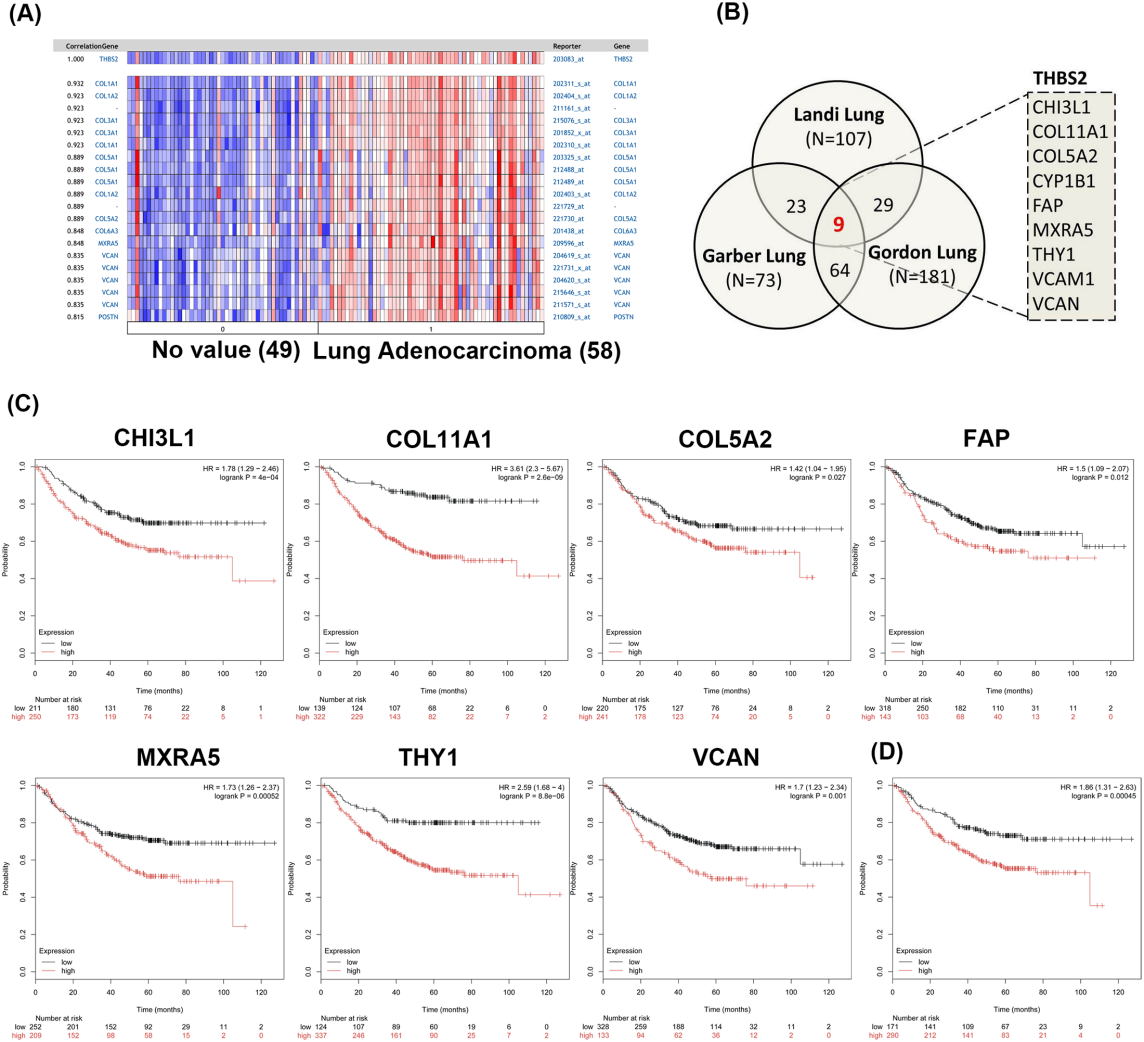
Prognosis analysis of THBS2 coexpressed genes in lung adenocarcinoma. (A) THBS2 coexpressed gene pattern in lung adenocarcinoma (Landi lung dataset). (B) 9 genes (CHI3L1, COL11A1, COL5A2, CYP1B1, FAP, MXRA5, THY1, VCAM1 and VCAN) were consistently identified as top 5% genes by co-expression score with Oncomine in lung AC datasets. (C) Kaplan-Meier survival curve (Kaplan-Meier plotter) showing the association of CHI3L1, COL11A1, COL5A2, FAP, MXRA5, THY1 and VCAN with progression-free survival in lung AC. (D) Kaplan-Meier survival curve showing the progression-free survival in lung AC with both high THBS2 and CYP1B1 expression, or both low THBS2 and CYP1B1 expression. AC, adenocarcinoma.

A previous study showed that a cluster of functional related genes were frequently coexpressed in different conditions, including in cancer formation [30]. To investigate the role of overexpressed THBS2 in pulmonary AC, gene signatures coexpressed with THBS2 were identified by microarray coexpression analysis. The top 5% of genes within the coexpression score from all three datasets were collected as a coexpressed list for analysis (Fig 3A). 98 genes were identified as coexpressed with THBS2 based on their presence in at least two of the three datasets mentioned above (S5 Table), and nine genes, CHI3L1, COL11A1, COL5A2, CYP1B1, FAP, MXRA5, THY1, VCAM1, and VCAN, were consistently overexpressed in three THBS2 coexpressed lists (Fig 3B and Table 3). To evaluate the significance of these nine genes in clinical outcomes, the association of their expression levels with survival was assessed using the Kaplan–Meier plotter tool. The survival analysis showed that higher CHI3L1, COL5A2, COL11A1, FAP, MXRA5, THY1, and VCAN expression levels was associated with significantly worse survival rates in pulmonary AC (Fig 3C and Table 3). Additionally, lung AC patients with high expression of both THBS2 and CYP1B1 had a poorer prognosis (HR = 1.86) than those with only high expression of THBS2 (HR = 1.75) or high expression of CYP1B1 (HR = 0.81) (Fig 3D).

**Table 3:** Analysis of genes co-expressing with THBS2 appeared in three lung adenocarcinoma datasets.

Gene		Log-rank p	Hazard ratio	Samplessize	Probe
**CHI3L1**	Chitinase 3-like 1	0.0004 ***	1.7	461	209396_s_at
**COL11A1**	Collagen, type XI, alpha 1	2.6e-09 ***	3.61		204320_at
**COL5A2**	Collagen, type V, alpha 2	0.0275*	1.42		221729_at
**CYP1B1**	Cytochrome P450, family 1, subfamily B, polypeptide 1	0.1788	0.81		202437_s_at
**FAP**	Fibroblast activation protein, alpha	0.0117 *	1.5		209955_s_at
**MXRA5**	Matrix-remodelling associated 5	0.0005 ***	1.73		209596_at
**THY1**	Thy-1 cell surface antigen	8.8e-06 ***	2.56		213869_x_at
**VCAM1**	Vascular cell adhesion molecule 1	0.1411	0.78		203868_s_at
**VCAN**	Versican	0.001 **	1.7		204619_s_at

* indicated*p*<0.05.

** indicated *p*<0.01.

***indicated *p*<0.001.

**Fig 4:**
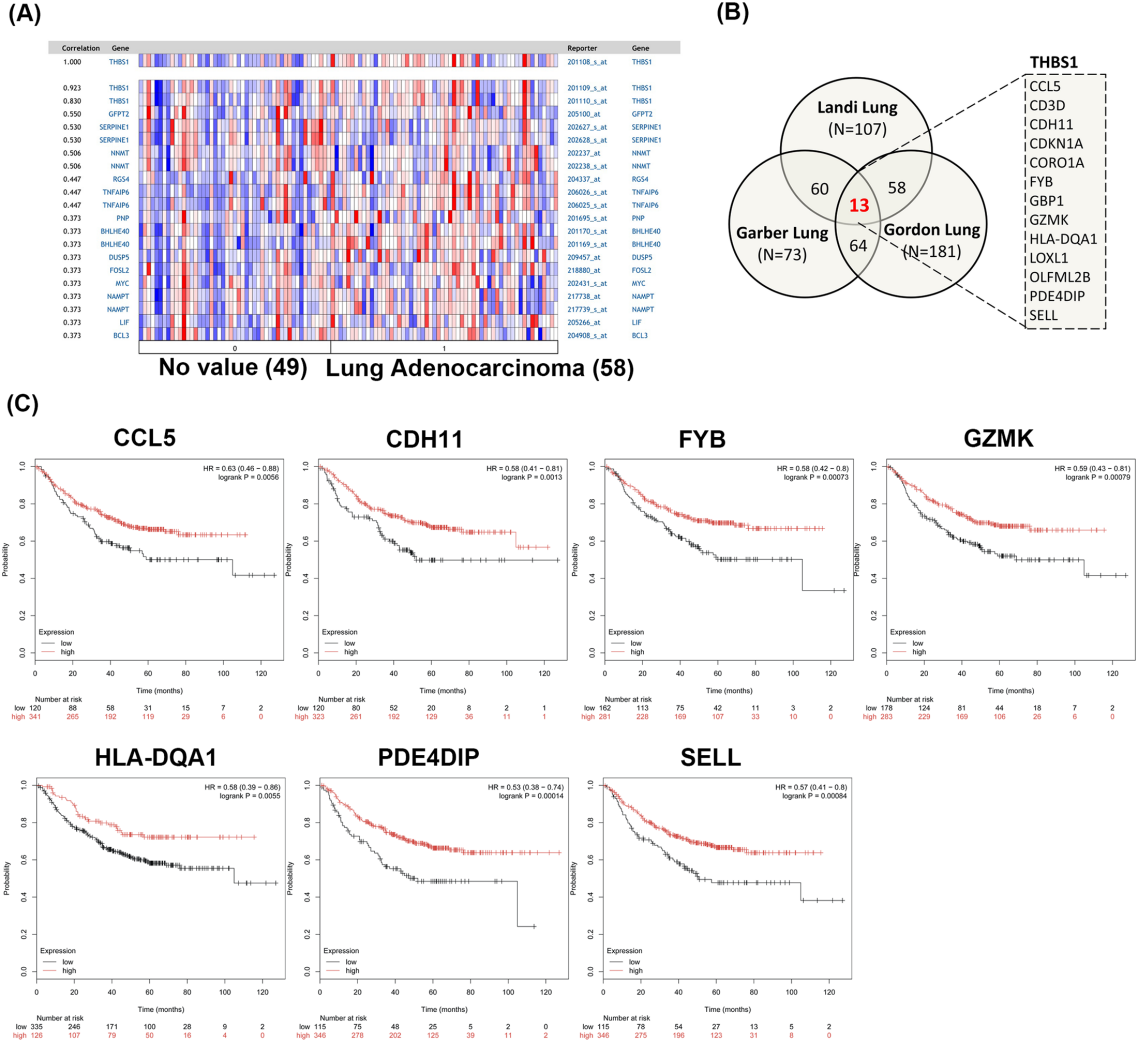
Prognosis analysis of THBS1 coexpressed genes in lung adenocarcinoma. (A) THBS1 coexpressed gene pattern in lung adenocarcinoma (Landi lung dataset). (B) 13 genes (CCL5, CD3D, CDH11, CDKN1A, CORO1A, FYB, GBP1, GZMK, HLA-DQA1, LOXL1, OLFML2B, PDE4DIP, and SELL) were consistently identified as top 5% genes by co-expression score with Oncomine in lung AC datasets. (C) Kaplan-Meier survival curve (Kaplan-Meier plotter) showing the association ofCCL5, CDH11, FYB, GZMK, HLA-DQA1, PE4DIP, and SELL with progression-free survival of in lung AC. AC, adenocarcinoma.

Using the same approach, genes coexpressed with THBS1 were identified to further study the distinct roles of THBS1 and THBS2 in pulmonary AC (Fig 4A and 4B). Thirteen genes, CCL5, CD3D, CDH11, CDKN1A, CORO1A, FYB, GBP1, GZMK, HLA-DQA1, LOXL1, OLFML2B, PDE4DIP, and SELL, appeared in the top 5% of genes coexpressed with THBS1 in the three datasets (Fig 4B and Table 4). Pulmonary AC patients with a high expression level of 7 of 13 genes (CCL5, CDH11, FYB, GZMK, LA-DQA1, PDE4DIP, and SELL) had better survival rates than those with a low expression level (Fig 4C and Table 4), respectively. The data revealed the distinguishing signatures of THBS1- and THBS2-coexpressed genes in pulmonary AC. The findings suggest that THBS2 and its coexpressing genes may play a significant role in biological processes during cancer progression.

**Table 4:** Analysis of genes co-expressing with THBS1 appeared in three lung adenocarcinoma datasets.

Gene		Log-rank p	Hazard ratio	Samples size	Probe
**CCL5**	Chemokine (C-C motif) ligand 5	0.0056 *	0.63	461	204655_at
**CD3D**	CD3 antigen, delta	0.0732	0.73		213539_at
**CDH11**	Cadherin 11, type 2, OB-cadherin (osteoblast)	0.0013 *	0.58		236179_at
**CDKN1A**	Cyclin-dependent kinase inhibitor 1A	0.0453 *	1.38		202284_s_at
**CORO1A**	Coronin, actin binding protein, 1A	0.2154	1.22		209083_at
**FYB**	FYN binding protein	0.0007 **	0.58		227266_s_at
**GBP1**	Guanylate binding protein 1	0.1403	0.79		202270_at
**GZMK**	Granzyme K	0.0008 **	0.59		206666_at
**HLA-DQA1**	Major Histocompatibility Complex, Class II, DQ Alpha 1	0.0055 *	0.58		213831_at
**LOXL1**	Lysyl oxidase-like 1	0.0108 *	1.52		203570_at
**OLFML2B**	Olfactomedin-like 2B	0.0002 *	1.83		213125_at
**PDE4DIP**	Phosphodiesterase 4D Interacting Protein	0.0001 ***	0.53		214099_s_at
**SELL**	Selectin L	0.0008 **	0.57		204563_at

* indicated*p*<0.05.

** indicated *p*<0.01.

***indicated *p*<0.001.

#### Differential gene signature coexpressed with THBS2 in breast and gastric cancer

To further explore the significance of genes coexpressed with THBS2 in lung AC, the gene signatures in breast cancer and gastric cancer with different prognostic characteristics were evaluated. The survival analysis indicated that increased THBS2 expression levels were associated with poor survival rates in breast cancer (S1A Fig). Genes coexpressed with THBS2 in breast cancer were examined (S1B Fig). Thirty-two genes (ADAM12, AEBP1, ASPN, ATXN10, BGN, COL10A1, COL1A1, COL5A2, COL6A2, COL8A2, COMP, CTSK, DCN, DPYL3, EMILIN1, FAP, FBLN1, FBN1, GLT8D2, ISLR, ITGBL1, LOXL1, LUM, MFAP5, MMP2, MXRA5, NID2, OLFML2B, RNF144A, SPARC, SPON1, and VCAN) were consistently coexpressed with THBS2 in breast cancer (S1C Fig). Four genes (COL5A2, FAP, MXRA5, and VCAN) were coexpressed with the THBS2 gene in lung AC. On the other hand, decreased THBS2 expression predicted a poor survival rate in patients with gastric cancer [31]. The THBS2 coexpressed genes were analyzed in gastric cancer. As revealed by the Kaplan–Meier plotter analysis, the survival of gastric cancer patients with high THBS2 expression was better than that of patients with low expression (S1D Fig). Thirteen genes (BGN, COL12A1, COL1A1, CTHRC1, FAP, FN1, INHBA, PDPNPR, RX1, SFRP4, SULF1, SPOCK1, and THBS1) were identified that were coexpressed with THBS2 (S1E and S1F Fig). These results suggested that THBS2 may have an oncogenic function in lung AC and breast cancer, with prediction of poor survival by overexpression. In contrast, THBS2 displays an anti-angiogenic function in gastric cancer since overexpression of THBS2 is associated with good prognosis. Several genes (COL5A2, MXRA5, and VCAN) were consistently coexpressed with THBS2 in lung AC and breast cancer, suggesting that these genes might be important in cancer progression

### Downregulation of THBS2 and VCAN in A549 cells inhibited cell growth

**Fig 5:**
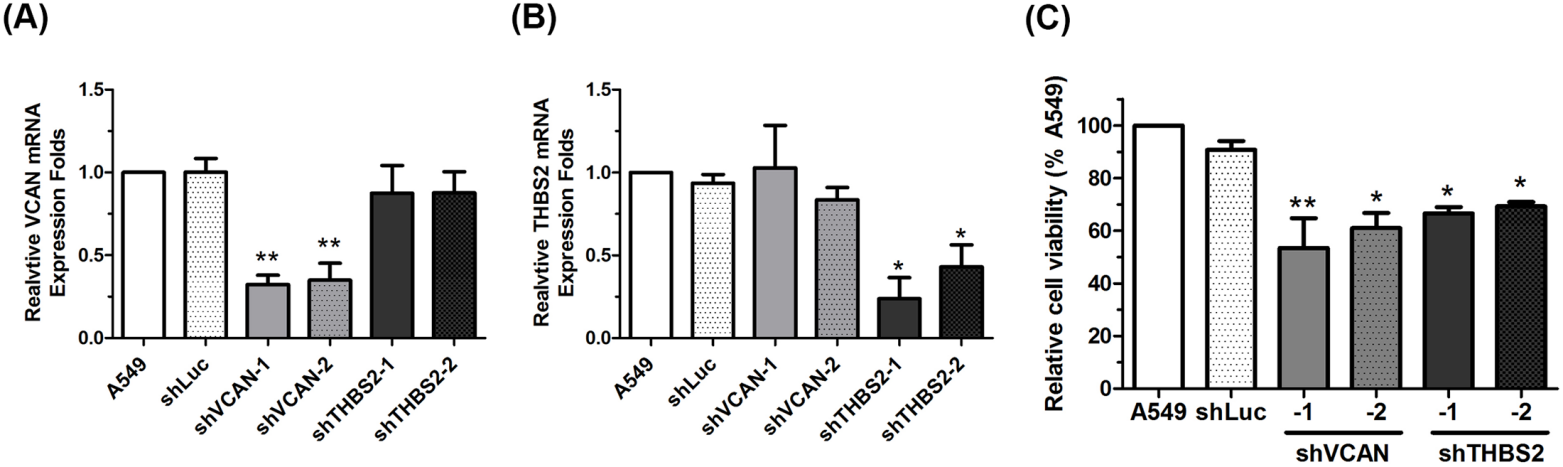
Down-regulation of VCAN or THBS2 decreased the cell proliferation in A549 cells. (A) mRNA expression level of VCAN was determined with real-time PCR. (B) mRNA expression level of THBS2 was determined by using real-time PCR. (C) The proliferation of A549 cells with VCAN or THBS2 knockdown expression were examined by WST-1 assay.* represent a *P* value < 0.05. ** represent a *P* value < 0.005.

Moreover, we investigated the significant role of THBS2 and its coexpressed gene VCAN in lung AC, the mRNA expression of THBS2 and VCAN was downregulated with shRNA (Fig 5A and 5B), and the cell growth of the A549 cells was examined. The cell proliferation of VCAN-knockdown A549 and THBS2-knockdown A549 cells were reduced when comparing with the control groups (Fig 5C). These results suggested that the gene signatures of lung cancer patients with increased THBS2 expression and a poor prognosis were more similar to those of breast cancer. COL5A2, MXRA5, and VCAN coexpressed with THBS2 may have significant oncogenic functions in both breast and lung cancers.

### Gene ontology and pathway enrichment analysis of THBS2 coexpressed genes identified the association of THBS2 with immune- and cancer-related processes in lung tumor development

**Fig 6:**
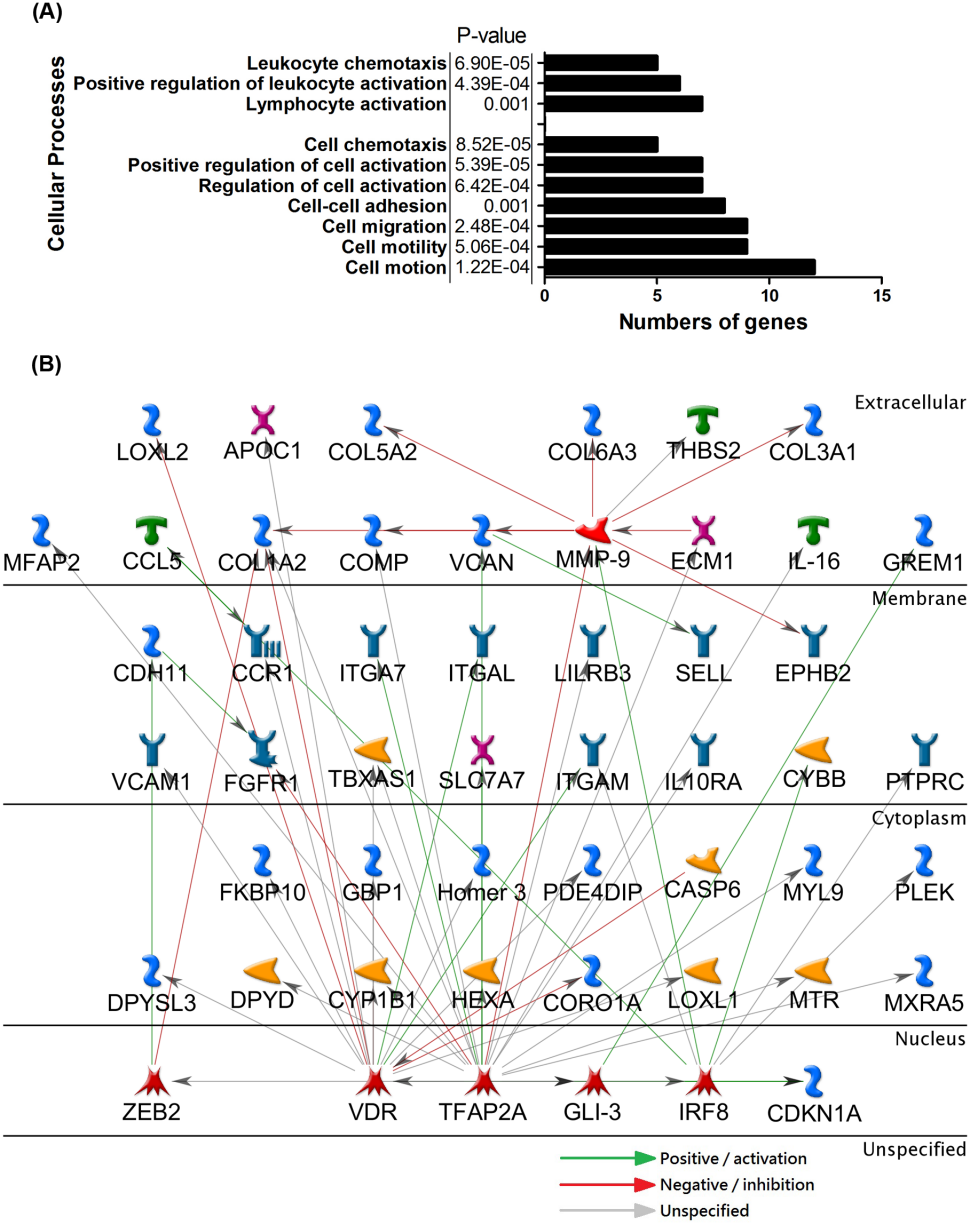
Gene ontology analysis and network construction of THBS2 coexpressed genes. (A) Gene ontology analysis of THBS2 coexpressed genes were conducted by using the DAVID (Database for Annotation, Visualization and Integrated Discovery) bioinformatics tool. P<0.001 for the pathway enrichment of THBS2 coexpressed genes compared with *Homo sapiens* transcriptome background. (B) The biological interactions of THBS2 coexpressed genes were analyzed by using Metacore. Red line, negative/ inhibition effects; Green line, positive/ activation effects. Gray line, unspecified effects.

To identify the mechanisms underlying the expression of THBS2 and its coexpressed genes, the gene ontology and pathway enrichment analysis were conducted using Database for Annotation, Visualization and Integrated Discovery (DAVID) bioinformatics tool [25]. Overall, 28 biological processes, 5 cellular constituents, 3 molecular function terms, and 4 Kyoto encyclopedia of genes and genomes (KEGG) pathways were indicated as being significantly enriched (S6 Table). Based on the results of the analysis, “system development” and “regulation of the immune process” showed the greatest marked enrichment among the biological process terms. Previous studies suggested that THBS2 seemed to be involved in many processes relating to system development and the inflammation response. This evidence supports the reliability of the results of the present analysis. Additionally, many terms related to immune regulation or cancer-related processes were enriched, such as cell motion, cell migration, and cell motility (Fig 6A). Extracellular matrix (ECM)-related terms took the most part in CC and KEGG pathways. To further investigate the functional connections of the THBS2-coexpressed genes, an interaction network analysis was performed using MetaCore. The network analysis revealed that TFAP2A, VDR, and MMP9 were strongly associated with 23, 21, and 11 THBS2-coexpressed genes, respectively, (23%, 21%, and 11% of the coexpressed gene list, respectively) (Fig 6B). Overall, the results suggest that the cluster of genes that was coexpressed with THBS2 may promote cancer progression through the induction of biological processes and immune modulation. The possible carcinogenic mechanism of the candidate target genes that were identified needs to be further verified in future studies.

## Discussion

The present study demonstrated that the mRNA expression of THBS2 was dramatically increased in several datasets of pulmonary AC and SCC and significantly associated with the clinical outcome in pulmonary AC. Most studies have focused on the role of THBS1 in lung cancer rather than other THBSs [32, 33]. Decreased expression of the THBS1 protein was associated with a poor prognosis in patients with NSCLC [32]. In contrast, THBS2 was reported to be significantly up-regulated in the protein expression signature of NSCLC [34]. Higher levels of THBS2 in serum from advanced NSCLC patients predicted worse median survival (9 months) compared to that (23.7 months) of patients with lower expression levels of THBS2 [18]. An immunohistochemical study showed that THBS2 expression levels were significantly correlated with the tumor size and stage. High levels of stroma-expressed THBS2 seemed to be associated with poor survival in pulmonary AC patients [14]. Based on the current evidence, THBS2 may play a more important role than THBS1 in the development of pulmonary AC development and is strongly correlated with the clinical outcome. In contrast, THBS2 was reported to be markedly up-regulated in the tumor microenvironment of skin cancer patients and to have antiangiogenic effects [35]. The increase in THBS2 levels in lung cancer tissue may be viewed as resistant to THBS2-induced anti-angiogenic effect in the subtype of lung AC [36]. In contrast, the survival analysis indicated that overexpression of THBS2 in breast cancer was associated with a poor prognosis. This finding suggests that THBS2 may have an oncogenic property in lung AC and breast cancer, with both types of cancer sharing the same coexpressed genes, including COL5A2, MXRA5, and VCAN. However, THBS2 exhibited an antiangiogenic function in gastric cancer as overexpression of THBS2 is correlated with improved clinical outcomes. The gene signatures in gastric cancer and lung AC also differed.

Cancer cells not only activate tumor angiogenesis, they also modulate the local immune response by releasing extracellular signals, thereby creating an environment suitable for tumor growth. The presence of local inflammatory cells and cancer cells in the tumor microenvironment is a key biomarker of cancer progression [37]. THBS1 and THBS2 were reported to function as negative regulators of the inflammatory response by inducing CD47-mediated apoptosis of T cells or inhibiting T-cell proliferation [5, 38, 39]. The interaction of THBS and CD47 was reported to activate thymus-derived CD4^+^ CD25^+^ T-regulatory cells in response to inflammation [40]. THBS2 may be a multi-function molecule, inhibiting both tumor-associated angiogenesis and local immune responses. The present study demonstrated that THBS2 and its coexpressed genes were involved not only in tumor-related ECM proteins, but also immune-related bioprocesses. On the other hand, targeting tumor-host interactions has become an incentive for anticancer strategies in drug development instead of targeting cancer cells [41], including the immune checkpoint-blocking inhibitors [42]. Therefore, the clinical outcome of THBS2-high NSCLC patients with immunotherapy targeting PD1 or the CTLA4 pathway deserves further evaluation in the future.

The analysis of molecular subtypes based on the modular gene signature is a potential strategy to further optimize targeted therapy for lung cancer [43, 44]. In three of the databases, CHI3L1, COL5A2, COL11A1, FAP, MXRA5, THY1, and VCAN were coexpressed with THBS2 and significantly correlated with the clinical outcome in pulmonary AC. Interestingly, these seven genes were all ECM or cell-surface proteins, and they all played a role in lung cancer progression. For example, CHI3L1 (YKL-40), a secreted inflammatory protein, is involved in the metastasis development of NSCLC cells through regulation of the epithelial-mesenchymal transition and migration/invasion enhancement [45], and correlated with a poor prognosis in NSCLC [46]. A recent study identified THY1 (CD90) as a potential cancer stem cell marker in NSCLC [47]. High expression levels of COL11A1, FAP, MXRA5, and VCAN predicted a poor prognosis in NSCLC patients [48–51]. Additionally, our results indicated that MMP9 played a major role in the THBS2-coexpressed gene network.MMP9 was reported to be involved in the progression of tumor metastasis and that it was associated with poor survival in NSCLC [52]. Altogether, these results demonstrate the potential role of THBS2 and its coexpressed genes in pulmonary AD with ECM features of consensus molecular subtype. The functional network analysis revealed functional connection of two nuclear proteins, TFAP-2 and VDR with other THBS2 coexpressed genes. The analysis also suggested that TFAP-2 and VDR may play predominant roles in the regulation of ECM and immune modulation during NSCLC development. It is interesting to note that 25-hydroxyvitamin D3 activates vitamin D receptor target gene expression and suppresses EGFR mutant non-small cell lung cancer growth [53]. However, the mechanisms underlying the activity of the THBS2-associated network need to be evaluated further in the future.

In conclusion, the present study indicated that THBS2 was overexpressed in NSCLC and the overexpression was significantly associated with a poor prognosis in human pulmonary AC. THBS2 and coexpressed genes were identified in this study and the gene products are highly involved in tumor-related bioprocesses. The findings of the present study can shed light on the specific role of THBS2 in the development and progression of lung cancer and clinical outcomes.

## Supporting Information

S1 Fig Analysis of survival and coexpressed genes of THBS2 in breast and gastric cancer. (A) Kaplan–Meier survival curve (Kaplan–Meier plotter) demonstrating the association of THBS2with progression-free survival in breast cancer. (B) THBS2 coexpressed gene pattern in breast cancer (Minn Breast 2 dataset). Groups: 0, no value (*n* = 1); 1, breast adenocarcinoma (*n* = 21); 2, breast carcinoma (*n* = 99). (C) Thirty-two genes (ADAM12, AEBP1, ASPN, ATXN10, BGN, COL10A1, COL1A1, COL5A2, COL6A2, COL8A2, COMP, CTSK, DCN, DPYL3, EMILIN1, FAP, FBLN1, FBN1, GLT8D2, ISLR, ITGBL1, LOXL1, LUM, MFAP5, MMP2, MXRA5, NID2, OLFML2B, RNF144A, SPARC, SPON1, and VCAN) consistently appeared in the top 5% genes identified by a coexpression score using Oncomine in breast cancer datasets. (D) The Kaplan–Meier survival curve (Kaplan–Meier plotter) illustrating the progression-free survival associated with THBS2 expression in gastric cancer. (E) THBS2 coexpressed gene pattern in breast cancer (Chen Gastric dataset). Groups: 0, no value (*n* = 29); 1. diffuse gastric adenocarcinoma (*n* = 13); 2, gastric adenocarcinoma (*n* = 15); 3, gastric intestinal type adenocarcinoma (*n* = 67); 4, gastric mixed adenocarcinoma (*n* = 8). (F) Thirteen genes (BGN, COL12A1, COL1A1, CTHRC1, FAP, FN1, INHBA, PDPNPR, RX1, SFRP4, SULF1, SPOCK1, and THBS1) consistently appeared in the top 5% genes identified by a coexpression score using the Oncomine in gastric cancer datasets differed.
(TIF)

S1 Table mRNA expression levels of THBS1 and THBS2 in colon cancer. (DOCX)

S2 Table mRNA expression levels of THBS1 and THBS2 in gastric cancer. (DOCX)

S3 Table mRNA expression levels of THBS1 and THBS2 in lung squamous cell carcinoma and small cell lung carcinoma. (DOCX)

S4 Table mRNA expression levels of THBS1 and THBS2 in pancreatic cancer. (DOCX)

S5 Table THBS2 co-expressed genes with the cut-off for selection defined as an appearance in two datasets. (DOCX)

S6 Table GO and pathway enrichment analysis of THBS2 co-expressed genes. (DOCX)

S7 Table Reference of mRNA expression profile in cancers generated from Oncomine (Table 1 and S1–S4 Tables). (DOCX)
