# Supplementary figures and images for: Differential Expression Pattern of THBS1 and THBS2 in Lung Cancer: Clinical Outcome and a Systematic-Analysis of Microarray Databases
Source: PLoS One. 2016 Aug 11;11(8):e0161007. doi: 10.1371/journal.pone.0161007 (PMC4981437; doi:10.1371/journal.pone.0161007)

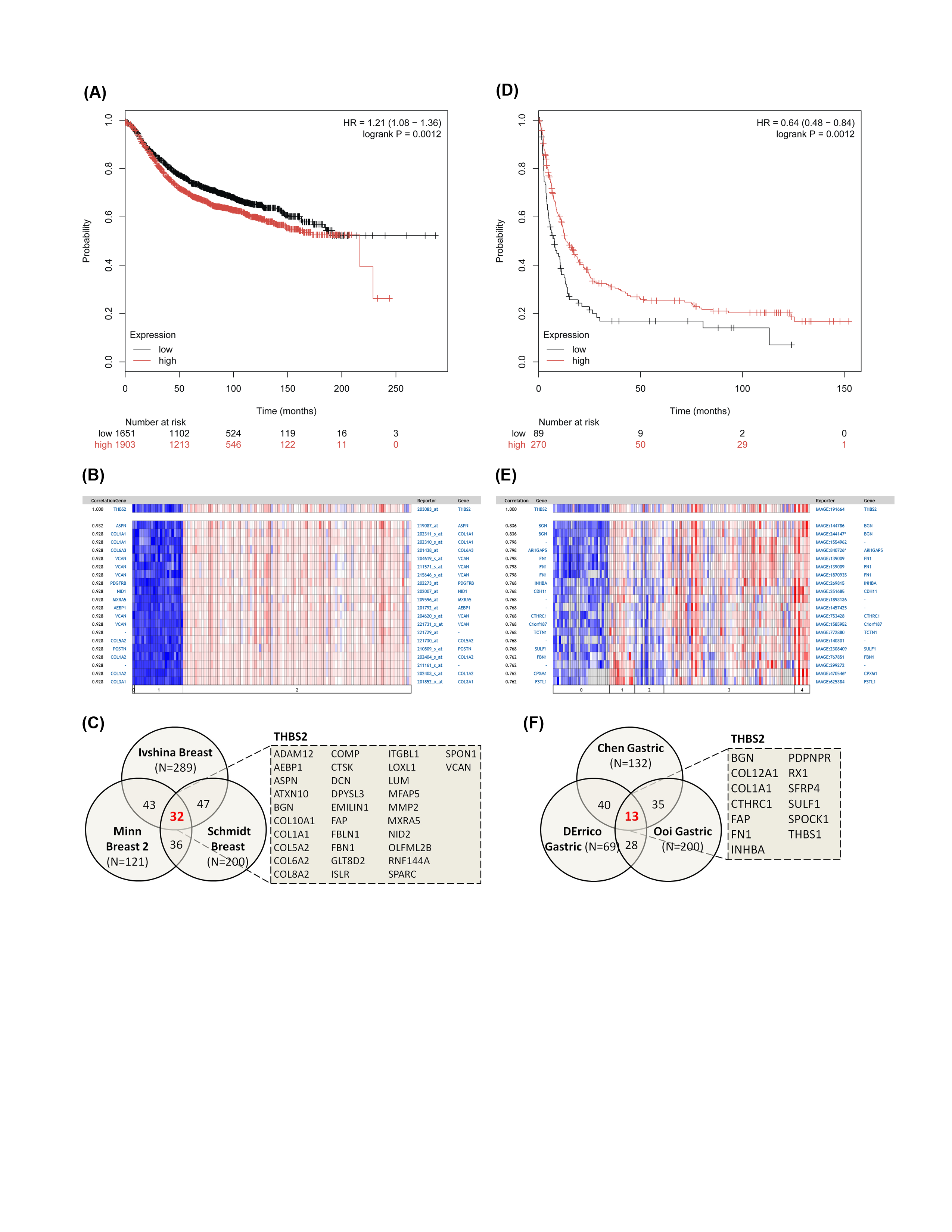

Supplement: S1 Fig — (A) Kaplan–Meier survival curve (Kaplan–Meier plotter) demonstrating the association of THBS2with progression-free survival in breast cancer. (B) THBS2 coexpressed gene pattern in breast cancer (Minn Breast 2 dataset). Groups: 0, no value (n = 1); 1, breast adenocarcinoma (n = 21); 2, breast carcinoma (n = 99). (C) Thirty-two genes (ADAM12, AEBP1, ASPN, ATXN10, BGN, COL10A1, COL1A1, COL5A2, COL6A2, COL8A2, COMP, CTSK, DCN, DPYL3, EMILIN1, FAP, FBLN1, FBN1, GLT8D2, ISLR, ITGBL1, LOXL1, LUM, MFAP5, MMP2, MXRA5, NID2, OLFML2B, RNF144A, SPARC, SPON1, and VCAN) consistently appeared in the top 5% genes identified by a coexpression score using Oncomine in breast cancer datasets. (D) The Kaplan–Meier survival curve (Kaplan–Meier plotter) illustrating the progression-free survival associated with THBS2 expression in gastric cancer. (E) THBS2 coexpressed gene pattern in breast cancer (Chen Gastric dataset). Groups: 0, no value (n = 29); 1. diffuse gastric adenocarcinoma (n = 13); 2, gastric adenocarcinoma (n = 15); 3, gastric intestinal type adenocarcinoma (n = 67); 4, gastric mixed adenocarcinoma (n = 8). (F) Thirteen genes (BGN, COL12A1, COL1A1, CTHRC1, FAP, FN1, INHBA, PDPNPR, RX1, SFRP4, SULF1, SPOCK1, and THBS1) consistently appeared in the top 5% genes identified by a coexpression score using the Oncomine in gastric cancer datasets differed. (TIF) [file pone.0161007.s001.tif]
