# Supplementary material for: Differential Expression Pattern of THBS1 and THBS2 in Lung Cancer: Clinical Outcome and a Systematic-Analysis of Microarray Databases
Source: PLoS One. 2016 Aug 11;11(8):e0161007. doi: 10.1371/journal.pone.0161007 (PMC4981437; doi:10.1371/journal.pone.0161007)
Supplement: S1 Table — (DOCX) [file pone.0161007.s002.docx]

**S1 Table. mRNA expression levels of THBS1 and THBS2 in colon cancer**

| Gene | P-Value  (Cancer/Normal) | Fold Change  (Cancer/Normal) | Ranking  (Top%) | Dataset | #Samples | Reference |
| --- | --- | --- | --- | --- | --- | --- |
| Colon Adenoma | | |  |  |  |  |
| THBS1 | 2.18E-8 | -2.409 | 4 | Sabates-Bellver | 64 | 9 |
|  | 7.11E-9 | -2.027 | 4 | Skrzypczak | 105 | 10 |
| THBS2 | 1.34E-8 | 3.475 | 1 | Skrzypczak 2 | 40 | 10 |
|  | 3.55E-11 | 5.610 | 2 | Kaiser | 105 | 12 |
|  | 4.06E-14 | 3.201 | 3 | Ki | 123 | 13 |
|  |  |  |  |  |  |  |
| Colon Carcinoma | |  |  |  |  |  |
| THBS2 | 1.65E-14 | 68.346 | 1 | Skrzypczak 2 | 40 | 10 |
|  | 1.89E-10 | 7.892 | 2 | Skrzypczak | 105 | 10 |
|  |  |  |  |  |  |  |
| Rectal Adenocarcinoma | |  |  |  |  |  |
| THBS2 | 7.01E-36 | 17.098 | 1 | Gaedcke | 130 | 11 |
|  | | |  |  |  |  |
| Cecum Adenocarcinoma | |  |  |  |  |  |
| THBS2 | 1.04E-6 | 4.414 | 4 | Kaiser | 105 | 12 |
|  |  |  |  |  |  |  |
| Colon Mucinous Adenocarcinoma | | |  |  |  |  |
| THBS2 | 8.97E-8 | 9.505 | 1 | Kaiser | 105 | 12 |
|  | 4.58E-9 | 4.541 | 5 | TCGA | 237 | 14 |
|  |  |  |  |  |  |  |
| Colon Carcinoma Epithelia | | |  |  |  |  |
| THBS2 | 8.57E-12 | 14.809 | 1 | Skrzypczak 2 | 40 | 9 |
|  |  |  |  |  |  |  |
| Rectosigmoid Adenocarcinoma | | |  |  |  |  |
| THBS2 | 4.05E-5 | 6.316 | 5 | Kaiser | 105 | 14 |

All references in this table were listed in the S7 Table.
