# Supplementary material for: Differential Expression Pattern of THBS1 and THBS2 in Lung Cancer: Clinical Outcome and a Systematic-Analysis of Microarray Databases
Source: PLoS One. 2016 Aug 11;11(8):e0161007. doi: 10.1371/journal.pone.0161007 (PMC4981437; doi:10.1371/journal.pone.0161007)
Supplement: S2 Table — (DOCX) [file pone.0161007.s003.docx]

**S2 Table. mRNA expression levels of THBS1 and THBS2 in gastric cancer**

| Gene | P-Value  (Cancer/Normal) | Fold Change  (Cancer/Normal) | Ranking  (Top%) | Dataset | #Samples | Reference |
| --- | --- | --- | --- | --- | --- | --- |
| Gastric Mixed Adenocarcinoma | | |  |  |  |  |
| THBS1 | 1.34E-7 | 5.452 | 1 | DErrico | 69 | 15 |
| THBS2 | 3.86E-6 | 8.964 | 1 | Chen | 132 | 16 |
|  | 3.86E-5 | 3.625 | 2 | Cho | 90 | 17 |
|  |  |  |  |  |  |  |
| Diffuse Gastric Adenocarcinoma | | |  |  |  |  |
| THBS2 | 2.11E-9 | 3.641 | 1 | Chen | 132 | 16 |
|  | 1.18E-8 | 4.801 | 1 | Cho | 90 | 17 |
|  |  |  |  |  |  |  |
| Gastric Intestinal Type Adenocarcinoma | | |  |  |  |  |
| THBS2 | 3.50E-19 | 3.688 | 1 | Chen | 132 | 16 |
|  | 3.85E-5 | 3.821 | 2 | Cho | 90 | 17 |
|  |  |  |  |  |  |  |
| Gastric Cancer | |  |  |  |  |  |
| THBS2 | 3.18E-5 | 8.661 | 1 | Wang | 27 | 18 |
|  | 1.40E-6 | 2.728 | 2 | Cui | 160 | 19 |

All references in this table were listed in the S7 Table.
