# Supplementary material for: Differential Expression Pattern of THBS1 and THBS2 in Lung Cancer: Clinical Outcome and a Systematic-Analysis of Microarray Databases
Source: PLoS One. 2016 Aug 11;11(8):e0161007. doi: 10.1371/journal.pone.0161007 (PMC4981437; doi:10.1371/journal.pone.0161007)
Supplement: S3 Table — (DOCX) [file pone.0161007.s004.docx]

**S3 Table. mRNA expression levels of THBS1 and THBS2 in lung squamous cell carcinoma and small cell lung carcinoma**

| Gene | P-Value  (Cancer/Normal) | Fold Change  (Cancer/Normal) | Ranking  (Top%) | Dataset | #Samples | Reference |
| --- | --- | --- | --- | --- | --- | --- |
| Squamous cell carcinoma | | |  |  |  |  |
| THBS2 | 1.56E-5 | 17.529 | 2 | Bhattacharjee | 203 | 1 |
|  | 6.00E-5 | 3.375 | 1 | Yamagata | 31 | 8 |
|  | 4.86E-15 | 5.841 | 2 | Hou | 92 | 6 |
|  |  |  |  |  |  |  |
| Small Cell Lung Carcinoma | | |  |  |  |  |
| THBS1 | 2.52E-5 | -5.923 | 5 | Bhattacharjee | 203 | 1 |

All references in this table were listed in the S7 Table.
