# Supplementary material for: Differential Expression Pattern of THBS1 and THBS2 in Lung Cancer: Clinical Outcome and a Systematic-Analysis of Microarray Databases
Source: PLoS One. 2016 Aug 11;11(8):e0161007. doi: 10.1371/journal.pone.0161007 (PMC4981437; doi:10.1371/journal.pone.0161007)
Supplement: S4 Table — (DOCX) [file pone.0161007.s005.docx]

**S4 Table. mRNA expression levels of THBS1 and THBS2 in pancreatic** **cancer**

| Gene | P-Value  (Cancer/Normal) | Fold Change  (Cancer/Normal) | Ranking  (Top%) | Dataset | #Samples | Reference |
| --- | --- | --- | --- | --- | --- | --- |
| Pancreatic Ductal Adenocarcinoma | | |  |  |  |  |
| THBS2 | 1.28E-17 | 16.427 | 1 | Badea | 78 | 20 |
|  |  |  |  |  |  |  |
| Pancreatic Adenocarcinoma | | |  |  |  |  |
| THBS2 | 2.50E-6 | 23.439 | 2 | Logsdon | 27 | 21 |
|  | 6.98E-5 | 6.682 | 2 | Iacobuzio-Donahue | 36 | 22 |
|  |  |  |  |  |  |  |
| Pancreatic Carcinoma | | |  |  |  |  |
| THBS2 | 3.60E-5 | 7.718 | 2 | Segara | 17 | 23 |
|  |  |  |  |  |  |  |
| Pancreatitis | |  |  |  |  |  |
| THBS2 | 3.84E-5 | 11.641 | 1 | Logsdon | 27 | 21 |

All references in this table were listed in the S7 Table.
