# Supplementary material for: Differential Expression Pattern of THBS1 and THBS2 in Lung Cancer: Clinical Outcome and a Systematic-Analysis of Microarray Databases
Source: PLoS One. 2016 Aug 11;11(8):e0161007. doi: 10.1371/journal.pone.0161007 (PMC4981437; doi:10.1371/journal.pone.0161007)
Supplement: S5 Table — (DOCX) [file pone.0161007.s006.docx]

**S5 Table. THBS2 co-expressed genes with the cut-off for selection defined as an appearance in two datasets**

| Gene | Gene Name | No. of appearances |
| --- | --- | --- |
| ADAM12 | ADAM metallopeptidase domain 12 | 2 |
| AIF1 | allograft inflammatory factor 1 | 2 |
| AIM2 | absent in melanoma 2 | 2 |
| APOC1 | apolipoprotein C-I | 2 |
| APOC2 | apolipoprotein C-II | 2 |
| ARL4C | ADP-ribosylation factor-like 4C | 2 |
| BCL2A1 | BCL2-related protein A1 | 2 |
| C3AR1 | complement component 3a receptor 1 | 2 |
| CASP6 | caspase 6, apoptosis-related cysteine peptidase | 2 |
| CCL5 | chemokine (C-C motif) ligand 5 | 2 |
| CCR1 | chemokine (C-C motif) receptor 1 | 2 |
| CD37 | CD37 molecule | 2 |
| CD3D | CD3d molecule, delta (CD3-TCR complex) | 2 |
| CDH11 | cadherin 11, type 2, OB-cadherin (osteoblast) | 2 |
| CDKN1A | cyclin-dependent kinase inhibitor 1A (p21, Cip1) | 2 |
| CHI3L1 | chitinase 3-like 1 | 3 |
| CILP | cartilage intermediate layer protein, nucleotide pyrophosphohydrolase | 2 |
| CNN1 | calponin 1, basic, smooth muscle | 2 |
| COL11A1 | collagen, type XI, alpha 1 | 3 |
| COL1A2 | collagen, type I, alpha 2 | 2 |
| COL3A1 | collagen, type III, alpha 1 | 2 |
| COL5A2 | collagen, type V, alpha 2 | 3 |
| COL6A3 | collagen, type VI, alpha 3 | 2 |
| COMP | cartilage oligomeric matrix protein | 2 |
| CORO1A | coronin, actin binding protein, 1A | 2 |
| CTSK | cathepsin K | 2 |
| CYBB | cytochrome b-245, beta polypeptide | 2 |
| CYP1B1 | cytochrome P450, family 1, subfamily B, polypeptide 1 | 3 |
| DIO2 | deiodinase, iodothyronine, type II | 2 |
| DOCK2 | dedicator of cytokinesis 2 | 2 |
| DOK5 | docking protein 5 | 2 |
| DPYD | dihydropyrimidine dehydrogenase | 2 |
| DPYSL3 | dihydropyrimidinase-like 3 | 2 |
| ECM1 | extracellular matrix protein 1 | 2 |
| EPB41L2 | erythrocyte membrane protein band 4.1-like 2 | 2 |
| EPHB2 | EPH receptor B2 | 2 |
| EVI2B | ecotropic viral integration site 2B | 2 |
| FAP | fibroblast activation protein, alpha | 3 |
| FCER1G | Fc fragment of IgE, high affinity I, receptor for; gamma polypeptide | 2 |
| FGFR1 | fibroblast growth factor receptor 1 | 2 |
| FKBP10 | FK506 binding protein 10, | 2 |
| FMOD | fibromodulin | 2 |
| FYB | FYN binding protein (FYB-120/130) | 2 |
| FZD1 | frizzled homolog 1 | 2 |
| GBP1 | guanylate binding protein 1, interferon-inducible, 67kDa | 2 |
| GLI3 | GLI family zinc finger 3 | 2 |
| GREM1 | gremlin 1, cysteine knot superfamily, homolog | 2 |
| GZMK | granzyme K (granzyme 3; tryptase II) | 2 |
| HEXA | hexosaminidase A | 2 |
| HLA-DQA1 | HLA class II histocompatibility antigen, DQ alpha 1 | 2 |
| HOMER3 | homer homolog 3 | 2 |
| IL10RA | interleukin 10 receptor, alpha | 2 |
| IL16 | interleukin 16 | 2 |
| IL1RAP | interleukin 1 receptor accessory protein | 2 |
| IRF8 | interferon regulatory factor 8 | 2 |
| ITGA7 | integrin, alpha 7 | 2 |
| ITGAL | integrin, alpha L (antigen CD11A (p180), lymphocyte function-associated antigen 1; alpha polypeptide) | 2 |
| ITGAM | integrin, alpha M (complement component 3 receptor 3 subunit) | 2 |
| ITM2A | integral membrane protein 2A | 2 |
| KIAA1199 | KIAA1199 | 2 |
| LCP1 | lymphocyte cytosolic protein 1 (L-plastin) | 2 |
| LILRA2 | leukocyte immunoglobulin-like receptor, subfamily A (with TM domain), member 2 | 2 |
| LILRB3 | leukocyte immunoglobulin-like receptor, subfamily B (with TM and ITIM domains), member 3 | 2 |
| LOXL1 | lysyl oxidase-like 1 | 2 |
| LOXL2 | lysyl oxidase-like 2 | 2 |
| LRRC15 | leucine rich repeat containing 15 | 2 |
| MFAP2 | microfibrillar-associated protein 2 | 2 |
| MMP9 | matrix metallopeptidase 9 | 2 |
| MNDA | myeloid cell nuclear differentiation antigen | 2 |
| MTR | 5-methyltetrahydrofolate-homocysteine methyltransferase | 2 |
| MXRA5 | matrix-remodelling associated 5 | 3 |
| MYL9 | myosin, light chain 9, regulatory | 2 |
| NCKAP1L | NCK-associated protein 1-like | 2 |
| NKG7 | natural killer cell group 7 sequence | 2 |
| OLFML2B | olfactomedin-like 2B | 2 |
| PDE4DIP | phosphodiesterase 4D interacting protein | 2 |
| PLEK | pleckstrin | 2 |
| POSTN | periostin, osteoblast specific factor | 2 |
| PTPRC | protein tyrosine phosphatase, receptor type, C | 2 |
| SELL | selectin L | 2 |
| SEMA3A | sema domain, immunoglobulin domain (Ig), short basic domain, secreted, (semaphorin) 3A | 2 |
| SLC1A3 | solute carrier family 1 (glial high affinity glutamate transporter), member 3 | 2 |
| SLC7A7 | solute carrier family 7 (cationic amino acid transporter, y+ system), member 7 | 2 |
| SNCG | synuclein, gamma (breast cancer-specific protein 1) | 2 |
| SNX1 | sorting nexin 1 | 2 |
| SULF1 | sulfatase 1 | 2 |
| TARP | TCR gamma alternate reading frame protein | 2 |
| TBXAS1 | thromboxane A synthase 1 (platelet) | 2 |
| TFAP2A | transcription factor AP-2 alpha (activating enhancer binding protein 2 alpha) | 2 |
| THBS1 | thrombospondin 1 | 2 |
| THY1 | Thy-1 cell surface antigen | 3 |
| TP53TG1 | TP53 target 1 (non-protein coding) | 2 |
| TRIM22 | tripartite motif-containing 22 | 2 |
| TSPAN5 | tetraspanin 5 | 2 |
| VCAM1 | vascular cell adhesion molecule 1 | 3 |
| VCAN | versican | 3 |
| VDR | vitamin D (1,25- dihydroxyvitamin D3) receptor | 2 |
| ZEB2 | zinc finger E-box binding homeobox 2 | 2 |
