# Supplementary material for: Differential Expression Pattern of THBS1 and THBS2 in Lung Cancer: Clinical Outcome and a Systematic-Analysis of Microarray Databases
Source: PLoS One. 2016 Aug 11;11(8):e0161007. doi: 10.1371/journal.pone.0161007 (PMC4981437; doi:10.1371/journal.pone.0161007)
Supplement: S6 Table — (DOCX) [file pone.0161007.s007.docx]

**S6 Table. GO and pathway enrichment analysis of THBS2 co-expressed genes.**

| Category | Term | Function | Count | P-value | Fold enrichment | FDR |
| --- | --- | --- | --- | --- | --- | --- |
| GOTERM_BP_3 | |  |  |  |  |  |
|  | GO:0048731 | System development | 36 | 4.30E-08 | 2.536731917 | 6.03E-05 |
|  | GO:0002682 | Regulation of immune system process | 12 | 1.82E-05 | 5.117389927 | 0.025605 |
|  | GO:0048513 | Organ development | 26 | 1.84E-05 | 2.456131241 | 0.025805 |
|  | GO:0007626 | Locomotory behavior | 10 | 3.81E-05 | 5.992077622 | 0.053444 |
|  | GO:0042330 | Taxis | 8 | 4.88E-05 | 8.209146341 | 0.068543 |
|  | GO:0006935 | Chemotaxis | 8 | 4.88E-05 | 8.21E+00 | 6.85E-02 |
|  | GO:0050867 | Positive regulation of cell activation | 7 | 5.39E-05 | 10.35387827 | 7.56E-02 |
|  | GO:0030595 | Leukocyte chemotaxis | 5 | 6.90E-05 | 22.186882 | 9.69E-02 |
|  | GO:0006952 | Defense response | 14 | 7.12E-05 | 3.74E+00 | 0.099850 |
|  | GO:0009611 | Response to wounding | 13 | 7.26E-05 | 4.027128394 | 0.101839 |
|  | GO:0060326 | Cell chemotaxis | 5 | 8.52E-05 | 21.04909318 | 0.119546 |
|  | GO:0002684 | Positive regulation of immune system process | 9 | 8.98E-05 | 6.208598073 | 0.125946 |
|  | GO:0048583 | Regulation of response to stimulus | 12 | 1.01E-04 | 4.236978757 | 0.142147 |
|  | GO:0006928~ | Cell motion | 12 | 1.22E-04 | 4.15E+00 | 0.171550 |
|  | GO:0051239 | Regulation of multicellular organismal process | 17 | 1.22E-04 | 2.98E+00 | 0.171721 |
|  | GO:0051129 | Negative regulation of cellular component organization | 7 | 2.10E-04 | 8.093524562 | 2.94E-01 |
|  | GO:0009653 | Anatomical structure morphogenesis | 19 | 2.16E-04 | 2.606078204 | 0.303070 |
|  | GO:0016477 | Cell migration | 9 | 2.48E-04 | 5.353791092 | 0.348197 |
|  | GO:0030198 | ECM organization | 6 | 4.02E-04 | 9.472091932 | 0.562489 |
|  | GO:0002696 | Positive regulation of leukocyte activation | 6 | 4.39E-04 | 9.293373217 | 0.613930 |
|  | GO:0048870 | Cell motility | 9 | 5.06E-04 | 4.813180265 | 0.708650 |
|  | GO:0009887 | Organ morphogenesis | 12 | 5.35E-04 | 3.493253762 | 0.748743 |
|  | GO:0050865 | Regulation of cell activation | 7 | 6.42E-04 | 6.567317073 | 0.896921 |
|  | GO:0032879 | Regulation of localization | 12 | 1.02E-03 | 3.229828069 | 1.424946 |
|  | GO:0046649 | Lymphocyte activation | 7 | 1.25E-03 | 5.775278833 | 1.741834 |
|  | GO:0016337 | Cell-cell adhesion | 8 | 1.35E-03 | 4.758925415 | 1.872200 |
|  | GO:0002683 | Negative regulation of immune system process | 5 | 1.56E-03 | 9.890537761 | 2.165241 |
|  | GO:0048519 | Negative regulation of biological process | 22 | 1.96E-03 | 1.993390944 | 2.711661 |
| GOTERM_CC_2 | |  |  |  |  |  |
|  | GO:0031012 | ECM | 17 | 2.12E-10 | 8.10E+00 | 1.86E-07 |
|  | GO:0044421 | Extracellular region part | 26 | 2.48E-10 | 4.45E+00 | 2.18E-07 |
|  | GO:0005615 | Extracellular space | 17 | 2.89E-06 | 4.081281 | 0.00254 |
|  | GO:0044420 | ECM part | 8 | 6.56E-06 | 11.24455 | 0.005765 |
|  | GO:0009986 | Cell surface | 11 | 4.50E-05 | 5.198183 | 0.039606 |
| GOTERM_MF_3 | |  |  |  |  |  |
|  | GO:0030247 | Polysaccharide binding | 9 | 4.02E-06 | 9.53401 | 0.004265 |
|  | GO:0032403 | Protein complex binding | 8 | 1.80E-04 | 6.66E+00 | 0.190724 |
|  | GO:0046983 | Protein dimerization activity | 11 | 1.54E-03 | 3.310909 | 1.6174 |
| KEGG_PATHWAY | |  |  |  |  |  |
|  | hsa04512 | ECM-receptor interaction | 9 | 5.19E-07 | 11.84394 | 5.37E-04 |
|  | hsa04510 | Focal adhesion | 10 | 5.06E-05 | 5.50E+00 | 5.24E-02 |
|  | hsa04670 | Leukocyte transendothelial migration | 7 | 5.35E-04 | 6.557664 | 0.551839 |
|  | hsa04514 | Cell adhesion molecules (CAMs) | 7 | 9.68E-04 | 5.862154 | 0.99742 |
| GO, gene ontolgy; FDR, false discovery rate; BP; biological process; CC, cellular constituent; MF, molecular function; ECM, extracellular matrix; KEGG, Kyoto encyclopedia of genes and genomes. | | | | | | |
