# Supplementary material for: Differential Expression Pattern of THBS1 and THBS2 in Lung Cancer: Clinical Outcome and a Systematic-Analysis of Microarray Databases
Source: PLoS One. 2016 Aug 11;11(8):e0161007. doi: 10.1371/journal.pone.0161007 (PMC4981437; doi:10.1371/journal.pone.0161007)
Supplement: S7 Table — (DOCX) [file pone.0161007.s008.docx]

**S7 Table. Reference of mRNA expression profile in cancers generated from Oncomine (Table 1 and S1-4 Tables)**

| Dataset | References |
| --- | --- |
| Bhattacharjee | 1. Bhattacharjee A, Richards WG, Staunton J, Li C, Monti S, Vasa P, et al. Classification of human lung carcinomas by mRNA expression profiling reveals distinct adenocarcinoma subclasses. Proc Natl Acad Sci U S A. 2001;98(24):13790-5. |
| Landi | 1. Landi MT, Dracheva T, Rotunno M, Figueroa JD, Liu H, Dasgupta A, et al. Gene expression signature of cigarette smoking and its role in lung adenocarcinoma development and survival. PloS one. 2008;3(2):e1651. |
| Su | 1. Su LJ, Chang CW, Wu YC, Chen KC, Lin CJ, Liang SC, et al. Selection of DDX5 as a novel internal control for Q-RT-PCR from microarray data using a block bootstrap re-sampling scheme. BMC genomics. 2007;8:140. |
| Selamat | 1. Selamat SA, Chung BS, Girard L, Zhang W, Zhang Y, Campan M, et al. Genome-scale analysis of DNA methylation in lung adenocarcinoma and integration with mRNA expression. Genome Res. 2012;22(7):1197-211. |
| Stearman | 1. Stearman RS, Dwyer-Nield L, Zerbe L, Blaine SA, Chan Z, Bunn PA, Jr., et al. Analysis of orthologous gene expression between human pulmonary adenocarcinoma and a carcinogen-induced murine model. Am J Pathol. 2005;167(6):1763-75. |
| Hou | 1. Hou J, Aerts J, den Hamer B, van Ijcken W, den Bakker M, Riegman P, et al. Gene expression-based classification of non-small cell lung carcinomas and survival prediction. PloS one. 2010;5(4):e10312. |
| Wei | 1. Wei TY, Juan CC, Hisa JY, Su LJ, Lee YC, Chou HY, et al. Protein arginine methyltransferase 5 is a potential oncoprotein that upregulates G1 cyclins/cyclin-dependent kinases and the phosphoinositide 3-kinase/AKT signaling cascade. Cancer Sci. 2012;103(9):1640-50. |
| Yamagata | 1. Yamagata N, Shyr Y, Yanagisawa K, Edgerton M, Dang TP, Gonzalez A, et al. A training-testing approach to the molecular classification of resected non-small cell lung cancer. Clin Cancer Res. 2003;9(13):4695-704. |
| Sabates-Bellver | 1. Transcriptome profile of human colorectal adenomas. Mol Cancer Res. 2007 ;5(12):1263-75. |
| Skrzypczak and Skrzypczak 2 | 1. Modeling oncogenic signaling in colon tumors by multidirectional analyses of microarray data directed for maximization of analytical reliability. PLoS One. 2010 ;5(10). pii: e13091 |
| Gaedcke | 1. Mutated KRAS results in overexpression of DUSP4, a MAP-kinase phosphatase, and SMYD3, a histone methyltransferase, in rectal carcinomas. Genes Chromosomes Cancer. 2010;49(11):1024-34 |
| Kaiser | 1. Transcriptional recapitulation and subversion of embryonic colon development by mouse colon tumor models and human colon cancer. Genome Biol. 2007;8(7):R131. |
| Ki | 1. Whole genome analysis for liver metastasis gene signatures in colorectal cancer. Int J Cancer. 2007 ;121(9):2005-12. |
| TCGA | 1. Comprehensive molecular characterization of human colon and rectal cancer. Nature. 2012 ;487(7407):330-7 |
| DErrico | 1. Genome-wide expression profile of sporadic gastric cancers with microsatellite instability. Eur J Cancer. 2009 ;45(3):461-9. |
| Chen | 1. Variation in gene expression patterns in human gastric cancers. Mol Biol Cell. 2003; 14(8): 3208–3215 |
| Cho | 1. Gene expression signature-based prognostic risk score in gastric cancer. Clin Cancer Res. 2011 ; 17(7): 1850–1857. |
| Wang | 1. Upregulated INHBA expression is associated with poor survival in gastric cancer. Med Oncol. 2012;29(1):77-83 |
| Cui | 1. An integrated transcriptomic and computational analysis for biomarker identification in gastric cancer. Nucleic Acids Res. 2011;39(4):1197-207 |
| Badea | 1. Combined gene expression analysis of whole-tissue and microdissected pancreatic ductal adenocarcinoma identifies genes specifically overexpressed in tumor epithelia. Hepatogastroenterology. 2008;55(88):2016-27 |
| Logsdon | 1. Molecular profiling of pancreatic adenocarcinoma and chronic pancreatitis identifies multiple genes differentially regulated in pancreatic cancer. Cancer Res. 2003;63(10):2649-57 |
| Iacobuzio-Donahue | 1. Exploration of global gene expression patterns in pancreatic adenocarcinoma using cDNA microarrays. Am J Pathol. 2003;162(4):1151-62. |
| Segara | 1. Expression of HOXB2, a retinoic acid signaling target in pancreatic cancer and pancreatic intraepithelial neoplasia. Clin Cancer Res. 2005;11(9):3587-96. |
